# Management of capital liquidity in public hospitals under the epidemic situation of COVID-19

**DOI:** 10.3389/fpubh.2022.977221

**Published:** 2022-10-20

**Authors:** Xiaoqiao Hu, Wei Jin, Ailan Yang, Zhigang Hu

**Affiliations:** ^1^Department of Finance, The First College of Clinical Medicine Science, Yichang Central People's Hospital, China Three Gorges University, Yichang, China; ^2^Yichang Central People's Hospital at Zhijiang, Zhijiang, China; ^3^Department of Respiratory and Critical Care Medicine, The First College of Clinical Medicine Science, China Three Gorges University, Yichang, China; ^4^Department of Respiratory and Critical Care Medicine, Yichang Central People's Hospital, Yichang, China

**Keywords:** public hospitals, finance, liquidity, COVID-19, current ratio

## Abstract

The epidemic of COVID-19 has a great impact on the life and safety of people around the world. As the main force in the fight against COVID-19, the financial management of public hospitals will provide a strong guarantee for the diagnosis and treatment behavior of medical staff. The financial department needs to recognize the extent of the impact of COVID-19 on hospital finance, quantify and predict the potential risk factors, and develop reasonable financial management strategies. As an important part of assessing the financial health of public hospitals, the capital liquidity can be used as the focus direction of the hospital managers. In this study, we determine the effects of COVID-19 on the finance of public hospitals. Subsequently, we invested the conception, components, risk factors of capital liquidity in public hospitals. In addition, we provided some management strategies of capital liquidity in public hospitals under the epidemic of COVID-19. We deemed that good capital liquidity can ensure that medical staff have enough confidence and mentality to face the risk of death from COVID-19.

## Introduction

The data showed that more than 0.6 billion patients were diagnosed with COVID-19 with 6 million deaths worldwide (1). Despite the well control of COVID-19 in China, there are still intermittent, regional outbreaks of COVID-19. The epidemic of COVID-19 not only had a significant impact on people's lives and health, but also plunged global economic trade and cultural exchanges into recession and even stagnation. According to the estimates of World Bank, the global economy contracted by 5.2% in 2020, which was the deepest recession since World War II (2). As the main force in fighting COVID-19, public hospitals play a leading role and fully reflect its importance and public welfare in social health care. However, the financial situation of hospitals is severely tested while fighting COVID-19. During the epidemic period, public hospitals need to invest a lot of manpower, material and financial resources. After the end of the epidemic, public hospitals still need to face problems such as decreased patients and the shortage cash flow. About 62.5% of Chinese public hospitals have negative hospital medical surplus, which increased by 56.25% compared with 2019 (3). How to deal with the impact of COVID-19 on the financial management of public hospitals will be an important consideration to reflect the ability level of hospital managers.

Financial liquidity may measure the ability to pay cash obligations as they come due (4). Failure to meet these obligations can lead to the bankruptcy and financial distress. Maintaining appropriate levels of financial liquidity should determine the statutory functions of public hospitals and make their have better decision-making capacity to front the pandemic of COVID-19 (5). Herein, the study planned to exhibit the effect of COVID-19 on finance of public hospitals worldwide. Subsequently, we introduced the conception, components, risk factors of capital liquidity in hospitals. Additionally, this review also provided some management strategies for capital liquidity under the epidemic of COVID-19.

## Subsections relevant for the subject

### The impact of COVID-19 on finance

Public hospitals rely mainly on the income of outpatients and inpatients. The data from the USA showed that selective admission to patients increases the income by an average of 30% (6). However, the number of outpatients was significantly reduced due to strict quarantine measures and traffic control during the epidemic of COVID-19, meanwhile the cash flow also was significantly reduced. Additionally, hospitals need to dispatch medical staff to participate in the treatment of COVID-19. Some surgical services may be postponed or closed down, which affected the hospital's medical income. The epidemic of COVID-19 supplies resulted in the shortage and disruption of supply chain in the short term. Hospitals need to increase inventories and supply costs to cope with this crisis, which significantly increased hospital spending. COVID-19 had a great impact on the enterprise economy, which reduced business performance and the payment capacity of enterprise medical insurance. The economic recession affected people's economic income, and the purchasing power of commercial insurance might decline. In order to reduce costs, commercial insurance managers strictly control the medical reimbursement behavior. In addition, financial sources such as charities, government and public support inevitably fall in the recession. During the last economic recession, total charitable donations fell sharply with research and development costs spending down by about 10% (7). Although the State Council has allocated special funds to tackle these crisis, the timing and level of the funds still remain uncertain. It can be expected that COVID-19 brings negative results for medical income through both short-term and long-term effects.

A study from Brazil showed a decline in hospital admissions and the income in most hospitals in 2020 compared to 2019, and average ward occupancy rates declined from 85 to 73% (8). Average monthly income fell by 10%. Meanwhile, ventilator purchases significantly increased hospital funding expenditures. Another survey showed that medical income of inpatient fell by 39%, and outpatient income fell by 65% during the pandemic of COVID-19 (9). Research on children's hospitals showed that the pandemic of COVID-19 made medical income fall by $276 million in 2020 compared to 2019 (10). The American Hospital Association estimated that hospital losses reached $202.6 billion between March 1 and June 30 2020 (11). Until 2022, more than 60% of public hospitals in China occurs in financial crisis (3).

### Capital liquidity of public hospitals

The evaluation of the financial health of public hospitals should include the following six areas: profitability, fixed capital efficiency, capital structure, fixed assets life, working capital efficiency and liquidity (12). The operating ability of capital liquidity is considered as one of the most critical indicators to manage the finance of public hospitals under limited financial conditions (13). Poor capital liquidity affect the decision-making ability of public hospitals. Liquidity is currently measured by three indicators: cash ratio, current ratio and quick ratio (5). The cash ratio is defined as the ratio of quick assets to current liabilities, which is not affected by inventory sales and accounts. Current ratio refers to the ratio of current assets divided by current liabilities, which is used to measure the ability of current assets to be converted into cash to repay liabilities before short-term debts mature. Current assets include immediate cash, receivable accounts, prepayments and other assets that can be realized in the short term, but also include inventory, amortized expenses and other assets that can be realized in the long term. Rapid ratio refers to the ratio of quick assets to current liabilities. Rapid assets only include assets that can be realized in a short term and exclude some poor liquid assets such as deferred expenses, which can strictly reflect the ability and level of public hospitals to immediately repay debts. Among the three indicators, the cash ratio is the most reflective and conservative indicator of the capital liquidity of public hospitals. It is generally considered to be more than 20%. The current ratio is recommended above 2:1, and the quick ratio is 1:1 (14). Excessive liquidity indicates that the hospital's available operating funds have not been properly used. Compared with enterprises, public hospitals adopt a relatively conservative financial strategy management. The excess financial resources can be invested in new medical equipment and technology to improve the medical service capacity of hospitals themselves, and then improve the profitability of hospitals and the welfare treatment of employees. Low capital liquidity will lose the ability of public hospitals to fulfill their liabilities. Public hospitals must ensure sufficient capital liquidity to deal with the supplier's accounts without any payment delay, and have the ability to pay the medical staff and maintain the normal life of the employees.

### The components of capital liquidity

The assets of public hospitals were roughly divided into the following categories: cash, receivable accounts, inventory, and fixed assets. The liquidity of the four assets is different.

Cash is the asset with the strongest capital liquidity in public hospitals. In addition to the cash flow obtained from daily operating income, cash liquidity includes the raising and utilization ability of hospital's fund, and timely realization ability of funds. Not all funds can be immediately realized and controlled. For example, cash deposits are time deposits in the bank, and the hospital's cash disposal capacity will be limited before maturity. Therefore, the financial management department needs to classify the realization ability and duration of hospital funds, so as to better use the funds.

A large part of the receivable accounts of hospitals are medical insurance, which have a relatively high credit rating and strong liquidity. However, there are also some problems in reality. Not all health insurance institutions can timely settle the accounts, and even some health insurance cannot complete all the health insurance funds. Hospitals need to make good statistics on the payment capacity and payment cycle of medical insurance institutions at all levels and formulate corresponding countermeasures. For the part that cannot be realized, hospitals must retain its own ability to undertake. In addition, medical insurance institutions will strictly supervise the diagnosis and treatment behavior of medical staff, and the unreasonable diagnosis and treatment behavior may be punished. Hospital managers should give full consideration to such situations, restrain the medical behavior and standardize the diagnosis and treatment process of diseases.

The inventory liquidity of hospitals mainly includes the liquidity of drugs, medical consumables and other materials. Different inventories have different ability to realize them. The vast majority of inventories can be realized within a certain period. It is necessary for hospital managers to master the details of the realization cycles and capabilities of different inventories, and try to avoid the realization of inventories with high realization cost. However, the responsibilities of public hospitals limit that hospitals must have some treatment drugs and materials for rare diseases, and hospital management departments need to make good plans for these inventories. In the operation process, drugs and medical consumables may have the risks of expiration and accidental consumption. Meanwhile, storing and distributing drugs need a certain cost, and the resulting loss of inventory liquidity needs to be effectively controlled.

The liquidity of fixed assets depends on the frequency of effective use of medical equipment. In the service life of medical equipment, the higher the frequency of the use of medical equipment, the stronger its realization ability. According to their use efficiency, managers classify the fixed assets according to three states: overload operation, full load operation and under-full load operation. Overloaded medical equipment may lose its life. The under-full load medical equipment is idle, which requires maintenance costs and causes a waste of medical resources. Reasonable allocation of the liquidity of fixed assets is one of the key goals that hospital managers need to consider.

### Factors influencing capital liquidity of public hospitals

Public hospitals often assume special social responsibilities, and capital liquidity differed from other financial industry. The financial industry usually pursues the maximization of economic benefits, while public hospitals need to strike a balance between income and cost. Public hospitals usually pursue higher, better and more reasonable medical technology and equipment as far as possible while fully ensuring the quality of medical care (13). Previous study demonstrated that the capital liquidity of public hospitals mainly depends on the management of hospital finance, which is less affected by the market economy (15). Therefore, starting from the public hospitals themselves, we can better find out the influencing factors of capital liquidity.

Bem et al. performed a detailed analysis for the factors affecting the liquidity of funds in public hospitals based on the mathematical formulas of current ratio (CR = current assets/current liabilities) and Pearson's correlation coefficient (16). The first analysis showed no significant association between capital liquidity with hospital size or beds but positively with hospital healthcare income. In other words, the higher the medical income of public hospitals, the better the capital liquidity. In the second analysis, the researchers found that medical income per bed is positively associated with the capital liquidity of hospital medical income.The third analysis demonstrated that the profitability of public hospitals is clearly associated with the liquidity of funds.The stronger the profitability, the better the capital liquidity, and vice versa. The increase in profitability directly translated into increased capital liquidity. In the fourth analysis, the researchers observed a moderately negative correlation between capital liquidity and debt ratio. The higher the debt ratio, the worse the financial liquidity. Subsequently, Bem et al. further determined the relationships between the annual income per bed with the static liquidity ratios (current ratio and quick ratio) on the basis of 138 public hospitals in Poland (17). Two liquidity ratios were described by the following mathematical formulas: current ratio = (current assets)/(current liabilities); quick ratio = (current assets–inventories)/(current liabilities). The findings suggested that the average level of current ratio was 2.25, similar to the result of American hospitals (17). Pearson's correlation coefficient suggested that the increase in the annual income per bed improves liquidity measured both by current ratio and quick ratio. Meanwhile, the association between the annual income per bed and financial liquidity might be positive only up to a certain level, which has been estimated at 60,000–70,000 EUR per bed (17).

The other two studies assessed the factors influencing capital liquidity of public hospitals in USA and Iran by using the linear regression model (4, 18). The average current ratio and quick ratio is 2.06 and 2.07 in Washington hospitals, respectively (4). Cash conversion cycle can reflect the process by which liquidity is changing (4). Study from Iran demonstrated that financial liquidity was increased by the following measures: increasing their size of hospitals, adjusting capital structure with the optimal utilization, and promoting their service delivery (18).

Certainly, there may be other factors affecting financial liquidity, which included the proportion of payment performance of medical staff, the proportion of different states of fixed assets, the proportion of different payment methods of medical insurance, and the nature of hospitals, etc (16). In the future, more studies are warranted to explore the impact of these factors on the capital liquidity of public hospitals.

### Management strategy of capital liquidity under the epidemic of COVID-19

The diagnosis and treatment of COVID-19 are different from conventional diseases, which require more manpower and material resources to deal with the outbreak of COVID-19, and has a huge impact on the operation of surgery and outpatient services. Therefore, public hospitals must adjust their capital liquidity in a short time according to the characteristics of COVID-19. In addition, the long-term effects on funds will also be slowly shown, and public hospitals still need to make good plans in advance to prevent and deal with the re-outbreak of COVID-19. Therefore, public hospitals should make management strategies for capital liquidity, give full consideration of feasibility and potential benefits, and then formulate implementation priorities and processes. Here, we mainly explore the short-term management strategy of capital liquidity with reference to previous studies (4, 5, 16–18).

Firstly, public hospitals need to quickly prepare their short-term cash flow to cope with the demand of COVID-19 outbreak. Every public hospital should establish a firm partnership with the core banks and communicate timely, so as to ensure and even improve the level of cash liquidity.

Secondly, public hospitals must complete liquidity management of receivable accounts. The cost of diagnosis and treatment of COVID-19 is borne by the state in China, and the realization ability is strong, but the period of realization is uncertain. Public hospitals need to keep close contact with medical insurance payment institutions to ensure the timely realization of medical insurance receivable accounts.

Thirdly, public hospitals may seek timely and continuous suppliers to ensure the liquidity of inventory. Due to the impact of the outbreak and traffic control, the supply of drugs and supplies may have problems. Public hospitals must proactively contact suppliers to ensure the supply channels of drugs and supplies. The treatment of COVID-19 requires a lot of medical equipment, such as ventilator and oxygen machine, etc. After the outbreak of COVID-19, hospital demand of these equipment will decline significantly, but the cost of purchasing equipment is high. Public hospitals can actively communicate with suppliers and obtain new equipment by using the way of lease supplement, which make public hospitals and suppliers get the benefit from it.

In addition, the liquidity of fixed assets is also the focus of managers. Based on the disease characteristics of COVID-19, managers can divide the status of the required fixed assets. Fixed assets that need to be overloaded can be purchased more vigorously, while fixed assets that are not run at full capacity can be placed into the hospital sharing platform, and all departments can use them together to improve their use efficiency.

The use of donated materials is also a direction for hospital management to consider. In the early days of the outbreak of COVID-19 in Wuhan, people donated a large number of epidemic prevention materials. However, a large number of donated materials were left idle in warehouses due to the lack of experience, but the frontline workers of epidemic prevention had to face COVID-19 with their bare hands. Hospitals need to set up a management department of donated materials as soon as possible, sort out the distribution process of materials, and improve the liquidity of donated materials.

## Discussion

The negative effect of COVID-19 on the finance of public hospitals is enormous and long-standing worldwide. Our study provided a potential coping strategy in facing this crisis for hospital administrators. As the foothold of hospital operation, the financial management with appropriately financial liquidity is particularly important, especially in the epidemic of COVID-19. In addition to normal business, the duties and responsibilities of financial staff also includes timely inventory of existing assets, the enactment of short-term and long-term management strategy of capital liquidity, the management of special subsidy funds, the allocation of material, donation and condolence funds, etc. Once the emergency occurs, financial departments must effectively guarantee orderly implementation of the hospital's various materials and financial management work under the direction of contingency plan. The back-up plan of capital liquidity also is necessary.

Regrettably, we did not find any relevant study about capital liquidity for facing COVID-19 in our rapid review of the literature. The recommended level of capital liquidity ratio for public hospitals was not known under the epidemic of COVID-19 due to the lack of clinical practice. More studies are warranted to deeply explore the effect of capital liquidity on facing COVID-19 and affirm the optimal level of capital liquidity ratio.

Although it has been 3 years, the menace of COVID-19 still occurs worldwide. This study should provide insight and bring additional knowledge contribution to the management of COVID-19 and hospital finance worldwide. Controlling the epidemic of COVID-19 is not only a matter of medical staff, but financial management will also play an important role. Good capital liquidity can ensure that medical staff have enough confidence and mentality to face the risk of death from COVID-19. The management of liquidity is not just a cram, but a complete framework and process, and even need multiple management strategies to deal with different epidemics of COVID-19. In addition, summarizing experience and lessons, learning from each other to prevent the outbreak of COVID-19 at any time, and viewing the impact of COVID-19 on hospital finance with a long-term perspective can help public hospitals better perform their social responsibilities and protect the safety of people.

## Author contributions

XH prepared the concept and performed the analysis. WJ, AY, and ZH interpreted the results and supervised the study. All authors contributed to the article and approved the submitted version.

## Conflict of interest

The authors declare that the research was conducted in the absence of any commercial or financial relationships that could be construed as a potential conflict of interest.

## Publisher's note

All claims expressed in this article are solely those of the authors and do not necessarily represent those of their affiliated organizations, or those of the publisher, the editors and the reviewers. Any product that may be evaluated in this article, or claim that may be made by its manufacturer, is not guaranteed or endorsed by the publisher.
